# Mechanistic Insights into *Achyranthes Bidentata* Blume Water Extract in Alleviating NAFLD: PPARγ-Driven Synergistic Regulation of Lipid Metabolism and Macrophage Efferocytosis

**DOI:** 10.2174/0118715303385546250912094219

**Published:** 2025-10-02

**Authors:** Xiong Ying Li, Ru Meng Yao, Xiao Ming Zou, Yun Xu, Sha Ying Qiu, Ping Ping He, Li Jun Wang, Shan Shan Lei

**Affiliations:** 1 Department of Pharmacy, Jiangxi College of Traditional Chinese Medicine, Fuzhou, Jiangxi 344000, China;; 2 Department of Medicine, Zhejiang Academy of Traditional Chinese Medicine, Hangzhou, Zhejiang 310007, P.R. China

**Keywords:** *Achyranthes bidentata* Blume, Non-alcoholic fatty liver disease, PPARγ, macrophage efferocytosis, lipid metabolism, inflammation

## Abstract

**Introduction:**

*Achyranthes bidentata* Blume (AB), a traditional Chinese medicine, is used to treat Non-alcoholic fatty liver disease (NAFLD), although its precise mechanism of action remains unclear. This study investigated the effects of *Achyranthes bidentata* water extract (ABW) on NAFLD in mice induced by high-fat-fed.

**Methods:**

The chemical components of *Achyranthes bidentata* Blume water extract (ABW) were analyzed using ultra-performance liquid chromatography–mass spectrometry (UPLC-MS). After 12 weeks of ABW treatment, liver histopathology and hepatic lipid levels, UPLC-MS including total cholesterol (TC) and triglycerides (TG), were evaluated in NAFLD mice. Enzyme-linked immunosorbent assay, quantitative PCR, western blotting, and immunofluorescence were employed to assess changes in the PPARγ/LXRα signaling pathway and macrophage efferocytosis *in vivo* and *in vitro*.

**Results:**

*In vivo*, ABW treatment significantly reduced hepatic lipid accumulation (TC and TG) and alleviated hepatic lesions compared with the model group. ABW also upregulated genes associated with lipid metabolism and macrophage efferocytosis, while reducing the hepatic levels of inflammatory cytokines and the number of apoptotic cells. *In vitro*, ABW suppressed the expression of pro-inflammatory genes (*Il1b, Il6, Tnf,* and *Nos2*) and enhanced the expression of macrophage efferocytosis-related genes (*Pparγ*, *Gas6*, *Tyro3*, and *Ax*l) in ox-LDL-induced Raw 264.7 cells. UPLC-MS identified 501 compounds in ABW. Auto-docking analysis suggested that 4-dodecylbenzenesulfonic acid, isovanilic acid, and oleanolic acid are potential PPARγ activators present in ABW.

**Discussion:**

This study demonstrates that ABW ameliorates NAFLD by activating the PPARγ/LXRα signaling pathway and promoting macrophage efferocytosis.

**Conclusion:**

These findings provide a novel mechanistic insight into the therapeutic effects of multi-component herbal medicines for NAFLD.

## 1 INTRODUCTION

Non-alcoholic fatty liver disease (NAFLD) is a chronic liver disorder defined by hepatic steatosis that is not attributable to alcohol consumption or other known causes. The primary pathological features include lipid accumulation and hepatic steatosis [1]. Depending on the degree of liver damage, NAFLD can progress from simple steatosis to non-alcoholic steatohepatitis (NASH), liver fibrosis, cirrhosis, and even hepatocellular carcinoma. NAFLD is closely associated with metabolic disorders such as type 2 diabetes and cardiovascular diseases, and its impact on health should not be underestimated [1]. The pathogenesis of NAFLD involves multiple factors, including lipid toxicity, oxidative stress, insulin resistance, and adipokine imbalance [2].

The current global prevalence of NAFLD ranges from 20% to 30%, while the domestic prevalence is estimated at 12%-24%. With increasing dietary intake of fat and sugar, alongside sedentary lifestyles, NAFLD incidence continues to rise annually [3]. As research into the mechanisms underlying NAFLD remains incomplete, treatment strategies have primarily focused on lifestyle and dietary interventions, which often yield unsatisfactory outcomes. Thus, there is an urgent need for effective therapeutic options.

Dysregulated lipid and cholesterol metabolism plays a central role in the pathogenesis of NAFLD [4]. Peroxisome proliferator-activated receptors (PPARs) are nuclear hormone receptors involved in the regulation of fatty acid metabolism and gene expression. Among the three isoforms (α, β, and γ), PPARγ is especially relevant to cholesterol and lipid metabolism. PPARγ agonists have been shown to prevent hepatic steatosis and inflammation effectively, and to improve symptoms related to NAFLD [5]. Liver X receptor alpha (LXRα), a downstream target of PPARγ, regulates the transcription of metabolic genes involved in hepatic cholesterol homeostasis [5]. PPARγ activation upregulates LXRa, which subsequently increases the expression of ATP-binding cassette transporter A1/B1 (ABCA1/ABCB1), facilitating cholesterol efflux from hepatocytes [6]. Hence, activation of the PPARγ/LXRα signaling pathway may be a promising strategy for enhancing hepatic cholesterol clearance and ameliorating NAFLD [5].

Efferocytosis refers to the process by which phagocytes, such as macrophages, clear apoptotic cells. This essential physiological function prevents tissue necrosis. Impairment or deficiency of this phagocytic activity can lead to the accumulation of apoptotic cells, thereby contributing to various pathological conditions, including unresolved inflammation and disruption of tissue homeostasis. The tyrosine kinase receptor (TAM) family (including TYRO3, AXL, and MER) plays a key role in efferocytosis by recognizing phosphatidylserine (PS) on apoptotic cells via bridging molecules such as growth arrest-specific protein 6 (Gas6), protein S (ProS), and milk fat globule-epidermal growth factor 8 (MFGE8). These molecules facilitate the connection between apoptotic cells and phagocytes, promoting their recognition and clearance, which is crucial for tissue homeostasis and preventing inflammation and autoimmunity. Binding of the apoptotic cell marker component PS to TAM receptors through the bridging molecule can inhibit TLR4-mediated NF-κB-dependent TNF-α expression [7]. Impaired efferocytosis can contribute to the development of chronic inflammation in NAFLD [8], and restoring this process may offer a promising therapeutic strategy against NAFLD [9].

*Achyranthes bidentata* Blume (AB), the dried root of *Achyranthes* from the amaranth family, has a long history of medicinal use in China. First documented in the *Shennong Herbal Classic*, it is widely recognized for its ability to invigorate the liver and kidneys [10]. Modern research has demonstrated that AB possesses anti-inflammatory, anti-apoptotic, and anti-osteoporotic properties and can improve blood circulation [10]. Additionally, studies have shown that AB extract ameliorates LPS/D-GalN-induced acute liver injury in mice by reducing inflammation-related damage [11]. More recently, AB has been found to improve abnormalities in glucose and lipid metabolism, both of which are closely linked to the pathogenesis of NAFLD [12]. However, it remains unclear whether ABW can regulate cholesterol metabolism, especially by regulating the PPARγ pathway to alleviate NAFLD.

This study employed ApoE^−/−^ NAFLD mice fed a high-fat, high-cholesterol diet to induce NAFLD, and evaluated the efficacy of ABW by assessing hepatic lipid levels, histopathology, and inflammation. Mechanistic insights were further explored using quantitative PCR (qPCR), western blotting, tyramide signal amplification (TSA) techniques, and other technologies to investigate the effects of ABW on hepatic cholesterol metabolism and macrophage efferocytosis.

## 2 METHODS

### 2.1 Preparation of ABW

AB was purchased from Zhejiang University of Traditional Chinese Medicine Traditional Chinese Medicine Decoction Co., Ltd. ABW was prepared by decocting AB powder in boiling water at a ratio of 1:10 for 2 hours and then concentrated to 4 g/mL. Then, the decoction was diluted to the required concentration in the later stage based on the administered dosage.

### 2.2 Ultra-high-performance Liquid Chromatography- mass Spectrometry (UPLC-MS) Analysis of ABW

The ingredients of ABW were detected by UPLC-MS as described by previous research [13]. A 200 μL UPLC-MS aliquot of the 4 g/mL ABW solution was diluted into 1000 μL of an 80% (*v/v*) methanol solution in water. The mixture was vortexed for 10 minutes and subsequently centrifuged at 4°C, followed by centrifugation at 20,000 × g for 10 minutes. The supernatant was filtered through a 0.22-μm membrane filter and analyzed using UPLC-MS (UltiMate 3000 RS-Q Exactive, Thermo Fisher Scientific Corp., USA), equipped with a Welch AQ-C18 column (150 mm × 2.1 mm × 1.8 μm). HPLC-grade 0.1% formic acid (A) and methanol (B) were employed as the mobile phases.

### 2.3 Animal Experiment

#### 2.3.1 Animals

There were 18 male ApoE^-/-^ mice (8 weeks, 20 ± 2g) and six male control C57BL/6J mice (8 weeks, 20 ± 2g) supplied by the GemPharmatech Co., Ltd. (license number: SCXK (su) 2018-0008, Jiangsu, China). The environmental conditions remained stable, with a consistent temperature and humidity, as well as a 12/12 light/dark cycle.

#### 2.3.2 Grouping and Administration

After allowing an adaptation time of 7 days, the 18 ApoE^-/-^ mice were assigned to the following groups based on their body weight: ABW 2.4 g/kg group (ABWH), ABW 1.2 g/kg group (ABWL), and ApoE^-/-^ model group (MG). Another six C57BL/6J mice were assigned to the control group (CG). All ApoE^-/-^ mice were fed with a high-fat diet (Boaigang Biological Technology Co., Ltd., BIOG109C) for 12 weeks. Meanwhile, we used gavage for administration with 2.4 and 1.2 g/kg/day for 12 weeks, respectively. The MG and CG were administered with purified water under the same conditions.

#### 2.3.3 Measurements of Serum Lipid Profiles

After 12 weeks of ABW administration, the mice were fasted overnight, and blood was collected from the ophthalmic venous plexus. Samples were centrifuged at 3500 rpm for 10 minutes to obtain serum. Serum total cholesterol (TC, A111-1-1) and triglyceride (TG, A110-1-1) levels were measured using commercial kits (Nanjing Jiancheng Bioengineering Institute, China).

#### 2.3.4 Measurements of Inflammation Factors in the liver by Enzyme-Linked Immunosorbent Assay (ELISA)

The liver tissues were weighed and subsequently mixed with a volume of pre-cooled saline solution at a ratio of 1:9 (*w/v*). The samples were then homogenized using a homogenizer at 70 Hz for 10 s. Then, the homogenate solution was centrifuged at 12,000 rpm for 10 minutes at 4°C, and the resulting supernatant was prepared for analysis. The levels of tumor necrosis factor-α (TNF-α) (BEINGLAY, MU30030), interleukin-6 (IL-6) (BEINGLAY, MU30044), and IL-1β (BEINGLAY, MU30369) in the liver were quantified using mouse ELISA kits in accordance with the manufacturer's instructions. The protein content was detected using the BCA kit (KeyGENBioTECH, Jiangsu, China).

#### 2.3.5 The TUNEL Staining and Hematoxylin-eosin (H&E) Staining

Liver tissue sections (4 μm) were prepared to evaluate hepatic apoptosis. Apoptotic cells were detected using the TMR (red) TUNEL Cell Apoptosis Detection Kit (G1502, Servicebio, Wuhan, China), following the manufacturer’s instructions. An additional 4 μm liver sections were stained with hematoxylin and eosin (H&E) and examined under a light microscope (Nikon, Japan) to assess hepatic cell damage.

#### 2.3.6 The Multiplex Immunofluorescence by TSA of the Liver

The protein expressions of PPARγ, LXRα, ABCG1, CD68 (BOSTER, BA3638), and AXL (HUABIO, M1310-5) in liver tissues were assessed using the TSA multi-marker staining technique. The test kit (HKI000-5A) was obtained from Haoke Biotechnology Co., Ltd., and procedures were conducted according to the manufacturer’s instructions. Paraffin sections (4 μm thick) were deparaffinized with xylene, rehydrated through a graded ethanol series, and washed three times with PBS. Subsequently, the sections were treated with 0.3% hydrogen peroxide for blocking and then subjected to antigen retrieval using citrate buffer. Following a 30-minute blockade with 5% BSA, the first primary antibodies (1:200 dilution) were applied and incubated overnight at 4 °C. The sections were then washed and treated with an HRP-conjugated secondary antibody, followed by incubation at 37 °C for 1 hour. The sections were subsequently stained with different fluorescent dyes. After removing the antibody solution, the sections were incubated with the second primary antibody. Following incubation with all antibodies, the slides were mounted with a DAPI-containing medium for nuclear counterstaining and examined under a fluorescent microscope.

### 2.4 Cell Experiments

#### 2.4.1 Cell Culturing and CCK-8 Analysis

RAW264.7 cells were procured from OriCell Bioscience Inc. The cells were cultured in Dulbecco’s Modified Eagle Medium (DMEM) supplemented with 10% fetal bovine serum (FBS) and maintained in an incubator at 37 °C with 5% CO_2_. Next, 1 × 10^3^ RAW264.7 cells were seeded in a 96-well culture plate and treated with 0, 100, 200, or 400 μg/mL of ABW. After 24 hours of incubation, cell viability was assessed using CCK-8 kits (BOSTER, AR1160). Control cells were treated with an equivalent volume of solvent.

#### 2.4.2 The Efferocytosis Effect of Macrophages on Apoptotic Cells

Granulocytes were isolated from mouse blood using Percoll discontinuous density gradient centrifugation. The cells were collected, counted, and resuspended in complete DMEM culture medium, then plated in a 96-well plate at a density of 5 × 10^3^ cells/well and incubated at 37 °C under a 5% CO_2_ atmosphere. Next, 100 µL of TNF-α was added, diluted 1:2000 in each well, and allowed to react for 12 h. The supernatant was then discarded, and the wells were washed twice with 200 µL of PBS. Subsequently, 100 µL of PKH26 staining solution was added to each well and incubated at 37 °C in the dark for 30 minutes. RAW264.7 cells were incubated with ox-LDL and ABW for 24 h in a 6-well plate. The logarithmic growth phase RAW264.7 cells were then enumerated, resuspended in complete DMEM culture medium, and treated with the DID staining solution. The marked cells were incubated at 37 °C in the dark for 15 minutes in a 1.5-mL EP tube. In a 24-well plate, RAW264.7 cells and TNF-α-induced granulocytes were co-cultured at a 2:1 ratio. For each well, 200 µL of suspension containing 10,000 RAW264.7 cells and 100 µL of suspension containing 5,000 apoptotic granulocytes were added. The plate was gently agitated in a cross pattern and the cells were allowed to settle for approximately 30 minutes, followed by imaging under a fluorescent microscope.

### 2.5 Real-time Polymerase Chain Reaction (qPCR)

The liver tissues were crushed on an ice platform, and total RNA was extracted from the liver tissues using the mRNA extraction kit (Sparkjade^®^, Shandong, China). The expressions of *Pparγ*, *Lxrα*, *Abcg1*, *Abca1, Tyro3, Axl*, *Gas6*, and *Mfeg8* were determined as mentioned elsewhere [14]. The primer sequences used in the study are shown in Table **1**.

### 2.6 Western Blotting

The expression of PPARγ (Cohesion, CPA4778), LXRα (HUABIO, ET1704-51), and ABCG1 (Cohesion, CPA2410) proteins was detected by western blotting, following the methodology described previously [15]. Briefly, liver tissues were homogenized on ice, and total proteins were extracted using lysis buffer. Equal amounts of protein (20 μg) were separated by 10% SDS-PAGE and transferred onto 0.45-μm PVDF membranes. After blocking with 5% BSA at 37 °C for 1 h, the membranes were incubated with the corresponding primary antibodies (1:1000 dilution in PBST) overnight at 4 °C and then washed. The membranes were subsequently incubated with HRP-conjugated secondary antibodies (1:5000 dilution) for 2 h at room temperature. Protein bands were visualized using a chemiluminescent assay kit (Yamei, Shanghai, China), and protein expression levels were normalized to β-actin (Cohesion, CPA9121).

### 2.7 Statistical Analysis

Data were reported as the mean ± SD and analyzed using SPSS 16.0 software. Student t-tests or ANOVA were used for group comparisons, with *p* < 0.05 considered statistically significant.

## 3 RESULTS

### 3.1 ABW Decreased Lipid Deposition and Liver Histopathology Damage in ApoE^-/-^ Mice

Compared with the CG, ApoE^−/−^ mice exhibited yellowish, fatty liver morphology. After 12 weeks of ABW administration, both high-dose (ABWH) and low-dose (ABWL) treatments significantly improved liver appearance, restoring a healthier pink coloration compared with the MG (Fig. **1A**). Histological analysis (Figs. **1B** and **C**) revealed extensive hepatocyte ballooning, inflammatory infiltration, and necrosis in the MG, accompanied by a significantly elevated hepatic steatosis score (*p* < 0.01). ABWH treatment notably alleviated these pathological changes.

Hepatic TC and TG levels were significantly elevated in the MG compared with the CG (*p* < 0.01) (Figs. **1D** and **E**). ABWH markedly reduced both TG and TC levels (*p* < 0.01) after 12 weeks compared with the MG, indicating significant amelioration of lipid accumulation.

### 3.2 UPLC-MS Analysis of ABW Components

Using UPLC-MS, a total of 501 ingredients were identified from ABW in both positive and negative ion modes (Fig. **2**). The 20 most abundant compounds in the UPLC-MS chromatogram are listed in Table **2**. Notable bioactive components included ecdysterone, 4-dodecylbenzenesulfonic acid, palmitoleic acid, (15Z)-9,12,13-trihydroxy-15-octadecenoic acid, isovanillic acid, and 5α-androstan-3,6,17-trione.

### 3.3 Molecular Docking between ABW Active Components and PPARγ

PPARγ plays a crucial role in lipid metabolism and the efferocytic function of macrophages. PPARγ agonists are effective in preventing hepatic steatosis and inflammation, as well as in improving symptoms associated with NAFLD. Additionally, activation of macrophage PPARγ increases the expression of TAM family receptors (Tyro3, Axl, and MerTK), facilitating the clearance of apoptotic cells. Consequently, molecular docking was employed to identify the active components in ABW that target PPARγ.

Molecular docking results revealed that all eight selected ABW components exhibited binding energies below −5 kcal/mol with PPARγ, suggesting favorable interactions. Among them, 4-dodecylbenzenesulfonic acid, isovanilic acid, and oleanolic acid had the strongest affinities, with binding energies of −9.1, −7.8, and −7.5 kcal/mol, respectively (Table **3**). Binding interactions were identified between 4-dodecylbenzenesulfonic acid and GLN-286, GLN-284, and GLN-454. Isovanilic acid formed bonds with GLN-286 and GLU-460. Oleanolic acid formed bonds with ARG-280 and SER-342 (Fig. **3**). These results suggest that these three compounds are likely the key PPARγ-interacting constituents in ABW.

### 3.4 ABW Modulates the PPARγ/LXRα Pathway in ApoE^−/−^ Mice

Lipid accumulation and hepatic inflammation are inversely associated with the PPARγ/LXRα signaling axis. Immunofluorescence of PPARγ, LXRα, and ABCG1 is shown in Figs. (**4A** and **B**), revealing significantly reduced expression of PPARγ and LXRα proteins in ApoE^−/−^ mice compared with the CG (*p* < 0.01) (Figs. **4C** and **D**), especially in the co-expression of LXRα and ABCG1 (Fig. **4B**). ABWH treatment significantly restored the expression of PPARγ and LXRα proteins (*p* < 0.05, 0.01) (Figs. **4C** and **D**), while ABWL also improved PPARγ and ABCG1 levels (*p* < 0.05, 0.01) compared with the ApoE^−/−^ mice (Figs. **4A**, **C** and **E**).

The qPCR analysis confirmed that hepatic mRNA levels of *Pparγ*, *Lxrα*, *Abca1*, and *Abcg1* were significantly downregulated in ApoE^−/−^ mice (*p* < 0.05, 0.01) (Figs. **5A**-**D**), compared with the CG. ABWH treatments significantly reversed these gene expression changes of *Pparγ* and *Lxrα* (*p* < 0.05, 0.01) compared with the ApoE^−/−^ mice. Western blotting results further demonstrated dramatic upregulation of LXRα and ABCG1 protein levels following ABWH and ABWL administration (*p* < 0.05, 0.01) (Figs. **5E**-**G**).

### 3.5 ABW Attenuates Inflammation and Apoptosis in ApoE^−/−-^Mice

ELISA showed significantly elevated levels of hepatic inflammatory markers IL-6, IL-1β, and TNF-α in ApoE^−/−^ mice (*p* < 0.01), compared with the CG. These levels were significantly reduced by ABWH treatment (*p* < 0.05) (Figs. **6A**-**C**). In parallel, *tnf* mRNA expression was significantly increased in ApoE^−/−^ mice (*p* < 0.01) and significantly decreased after ABWH and ABWL administration (*p* < 0.01) (Fig. **6D**). Immunofluorescence revealed a significant reduction in apoptotic cells in ApoE^−/^ mice, indicating improved macrophage efferocytosis in the ABWH- and ABWL-treated groups (*p* < 0.01) (Figs. **6E** and **F**).

### 3.6 ABW Enhances Macrophage Efferocytosis in ApoE^−/−^ Mice

To evaluate ABW’s effect on hepatic macrophage efferocytosis, the expression of CD68 and AXL proteins was analyzed through immunofluorescence and western blotting. In ApoE^−/−^ mice, both proteins were significantly downregulated (*p* < 0.01) (Figs. **7A**-**C**). ABWL was significantly upregulated in expression (*p* < 0.01) compared with MG, with similar trends observed for ABWH. These findings suggest that ABW enhances macrophage efferocytosis, thereby contributing to the resolution of hepatic inflammation and improving liver function in ApoE^−/−^ mice with NAFLD.

The expressions of liver genes (*Axl, Gas6,* and *Mfeg8*) were detected through qPCR. The expression levels (Figs. **7D**-**F**) significantly decreased in the ApoE^−/−^ mice (*p* < 0.01), compared with the CG. ABWH dramatically increased *Axl and Mfeg8* expressions (*p* < 0.05, 0.01), and a certain trend of increasing *Gas6* levels was noted, compared with the MG. ABW may affect liver macrophage efferocytosis by upregulating the expressions of Axl, Gas6, and Mfeg8 in ApoE^−/−^ mice with NAFLD.

### 3.7 Effect of ABW on Macrophage Efferocytosis and the PPARγ/AXL/GAS6 Pathway in ox-LDL-induced Raw 264.7 Cells

*In vitro*, we first investigated the effects of ABW on cell proliferation using CCK8 assays. Compared with the control group, ABW significantly enhanced cell growth after 24 h at 800–1600 μg/mL, but had no significant effect at 50–400 μg/mL (Fig. **8A**). Next, compared with the ox-LDL group, ABW at 100, 200, and 400 μg/mL improved macrophage efferocytosis of apoptotic neutrophils (Figs. **8B** and **C**).

To determine whether ABW promotes macrophage efferocytosis, qPCR and western blotting were performed to measure the expression of PPARγ, GAS6, and TYRO3 in ox-LDL-induced macrophages. ABW at 400 μg/mL significantly upregulated the protein levels of PPARγ, TYRO3, and AXL (*p* < 0.05) (Figs. **8D**, **I**-**L**) and increased Gas6 mRNA expression (*p* < 0.01) (Fig. **8E**). Moreover, ABW treatment significantly decreased mRNA expression of the pro-inflammatory cytokines Il1b, Il6, and Nos2 (*p* < 0.05, 0.01) (Figs. **8F**-**H**).

## 4 DISCUSSION

NAFLD is a metabolic stress-induced liver condition caused by multifactorial etiologies or individual and synergistic effects. Its prevalence continues to rise globally, increasing with age [16]. Traditional Chinese medicine (TCM) has shown promising efficacy in the integrative treatment of NAFLD [17]. *Achyranthes bidentata* Blume, a well-known TCM, is recognized for its hepatoprotective properties [18] and its potential to treat liver-related diseases [11]. However, its role in the prevention and treatment of NAFLD remains unclear. In this study, we demonstrated that ABW significantly alleviates liver injury and inflammation, exerting therapeutic effects against NAFLD. Mechanistically, these effects appear to be mediated through the activation of the PPARγ/LXRα and PPARγ/TYRO3 pathways.

NAFLD pathogenesis involves multiple mechanisms, including lipotoxicity, inflammation, oxidative stress, and insulin resistance [19]. Both human and animal studies have reported dysfunction in cholesterol metabolism, lipid accumulation, and elevated pro-inflammatory cytokines (*e.g.*, IL-6, IL-1β, and TNF-α) in NAFLD [20]. Cholesterol accumulation contributes to hepatic inflammation [19] and disease progression [21]. Our results showed that ABW effectively reduced hepatic TC and TG levels and decreased levels of inflammatory cytokines (IL-6, IL-1β, and TNF-α) in ApoE^−/−^ mice. Relevant studies have also shown that AB effectively prevents and treats various liver diseases [22]. It effectively mitigates hepatic lipid accumulation and inflammation in NAFLD models [23], possibly by modulating IL-1β and other inflammatory mediators [24]. ABW’s ability to ameliorate liver tissue lesions and inflammatory damage in ApoE^−/−^ mice with NAFLD was consistent with findings from other studies.

The PPARγ/LXRα axis plays a central role in cholesterol metabolism and fatty liver disease [25]. PPARγ, a nuclear receptor and multifunctional transcription factor, regulates the expression of lipid metabolic genes in liver tissue [26] and also modulates cholesterol metabolism [27]. Upon activation, it induces LXRα, which in turn promotes cholesterol efflux [28]. Enhancing this pathway has been shown to improve lipid-induced tissue damage [29]. Key downstream targets of LXRα include ABCG1 and ABCA1, which mediate cholesterol efflux from tissues [30]. Notably, ABCA1 deficiency exacerbates cholesterol accumulation by activating sterol regulatory element-binding protein 2 (SREBP2), a key regulator of cholesterol synthesis and uptake [31]. Both ABCG1 and ABCA1 are directly regulated and transcriptionally activated by LXRα to promote cholesterol efflux in tissues [32]. In our study, ABW regulated the expression of PPARγ, LXRα, and ABCG1 in ApoE^−/−^ mice, suggesting a mechanism by which it improves cholesterol metabolism in NAFLD.

PPARγ, a ligand-activated member of the nuclear transcription factor superfamily, is also expressed in macrophages and plays a critical role in modulating their efferocytic function. Activation of macrophage PPARγ enhances the expression of TAM family receptors (Tyro3, Axl, MerTK) and bridging molecules such as GAS6 and MFGE8, facilitating the clearance of apoptotic cells. Loss of PPARγ impairs efferocytosis and increases pro-inflammatory cytokine production, while PPARγ agonists enhance phagocytosis of apoptotic cells by mouse macrophages [33]. Conversely, the PPARγ inhibitor GW9662 inhibits peritoneal macrophage phagocytosis and downregulates the expression of MFGE8, a bridging protein [34], and TAM receptors [35]. In liver disease, reduced AXL expression correlates with progression to cirrhosis [36]. GAS6 interacts with TAM receptors (Tyro3, Axl, and MerTK) to help macrophages clear apoptotic cells, which is crucial for tissue integrity and inflammation control [37]. GAS6 also encourages macrophages to adopt an anti-inflammatory state [38] and regulates immune responses by increasing macrophage activity, such as improving efferocytosis and reducing inflammation in obesity-related osteoarthritis [39]. MFGE8 is essential for efferocytosis, as it maintains immune balance by preventing autoimmune responses from necrotic cells [40]. MFGE8 inhibition worsens conditions such as NASH by increasing cell death and inflammation [41]. In our study, ABW decreased apoptotic cells and liver inflammatory factors, thereby activating the Tyro3/Axl/GAS6/MFGE8 pathway in ApoE^−/−^ mice. Moreover, ABW promoted macrophage efferocytosis, decreased levels of pro-inflammatory factors, and activated the PPARγ/TAM pathway in ox-LDL-induced RAW264.7 cells.

## CONCLUSION

This study demonstrates that ABW improves NAFLD by activating the PPARγ/LXRα pathway and the PPARγ/TAM axis, thereby modulating the interconnected network of lipid metabolism and macrophage efferocytosis. The mechanism is illustrated in Fig. (**9**). These findings provide novel insights into the mechanistic basis of multi-component herbal therapies for NAFLD. Molecular docking results suggested that 4-dodecylbenzenesulfonic acid, isovanilic acid, and oleanolic acid are key ABW components that bind PPARγ and may represent promising therapeutic candidates for NAFLD. Future research should validate the therapeutic efficacy of these compounds in NAFLD murine models and further elucidate their interactions with PPARγ using techniques such as surface plasmon resonance.

## LIMITATIONS

Despite the strong evidence, our study has limitations that should be considered when interpreting the results and suggest directions for future research. On the one hand, the ApoE^-/-^ mouse model is useful for studying lipid metabolism and inflammation but may not accurately reflect the complex metabolic issues of human NAFLD. Using diet-induced models that better simulate human metabolic syndrome would enhance the applicability of our findings. On the other hand, despite UPLC-MS revealing many constituents in ABW and molecular docking indicating possible PPARγ interactions, the extract's complexity poses a challenge. The exact bioactive compounds responsible for its effects are still unidentified. ABW's efficacy might stem from synergistic interactions among its components. Future research should focus on isolating key compounds and validating their individual and combined effects to identify the primary active ingredients.

## Figures and Tables

**Fig. (1):**
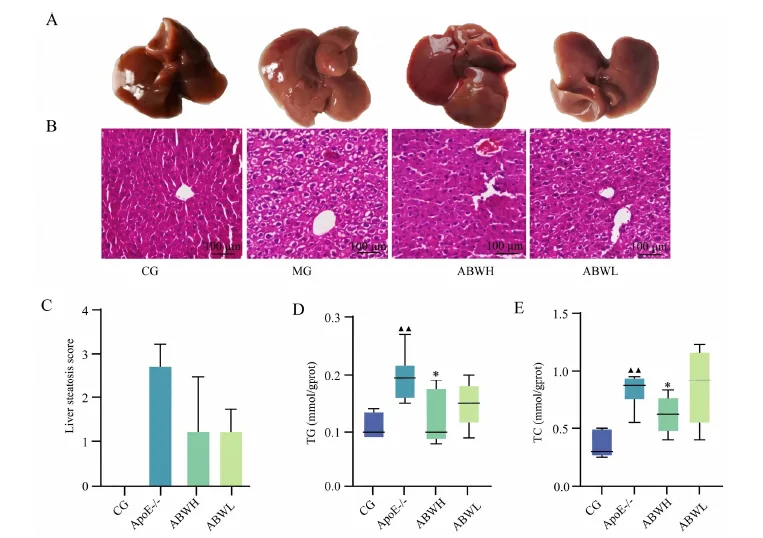
Effect of ABW on the liver histopathology and inflammatory factors in ApoE^-/-^ mice. (**A**) The liver's gross morphology. (**B**) Pathological images of the liver (H&E staining, magnification 200×). (**C**) Liver steatosis score. (**D–E**) Changes of the TC and TG levels in the liver. n=6. ^▲^*p* < 0.05, ^▲▲^*p* < 0.01 *vs*. CG. *p < 0.05, **p < 0.01 *vs*. ApoE^-/-^.

**Fig. (2):**
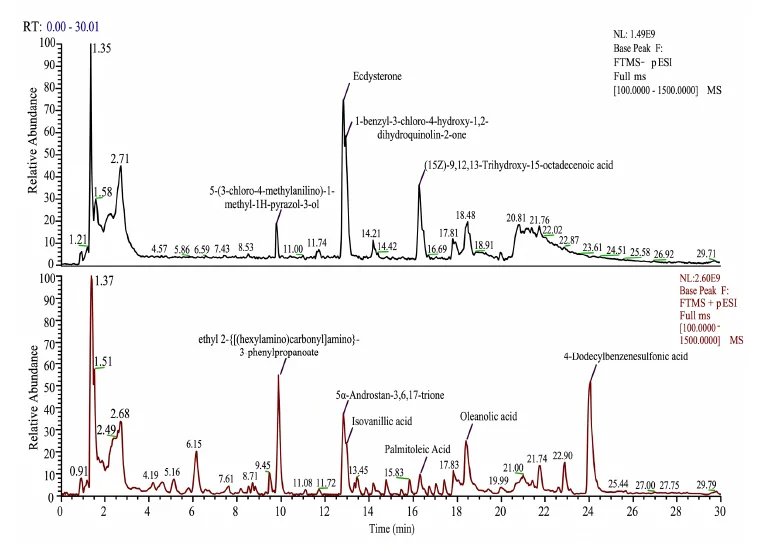
UPLC-MS analysis of ABW. The black line indicates the negative-ion mode, and the red line indicates the positive-ion mode.

**Fig. (3):**
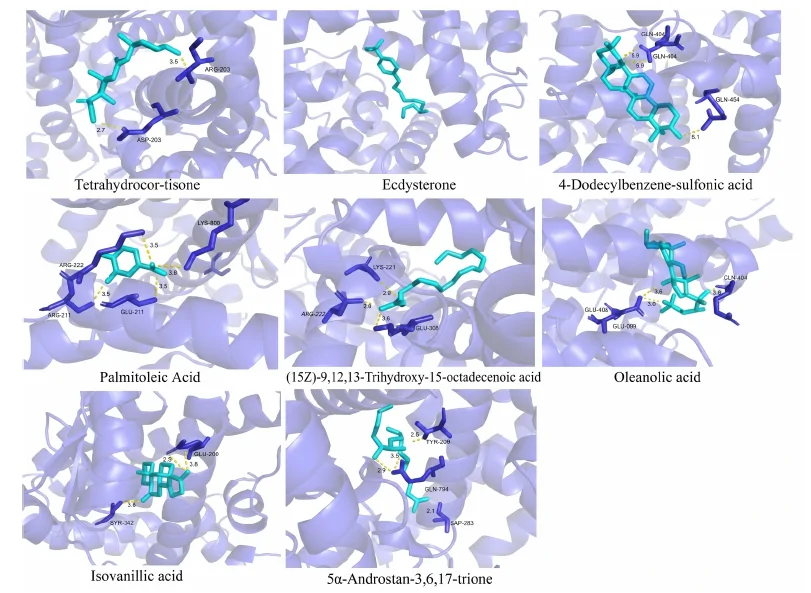
Molecular docking results between ABW components and PPARγ.

**Fig. (4):**
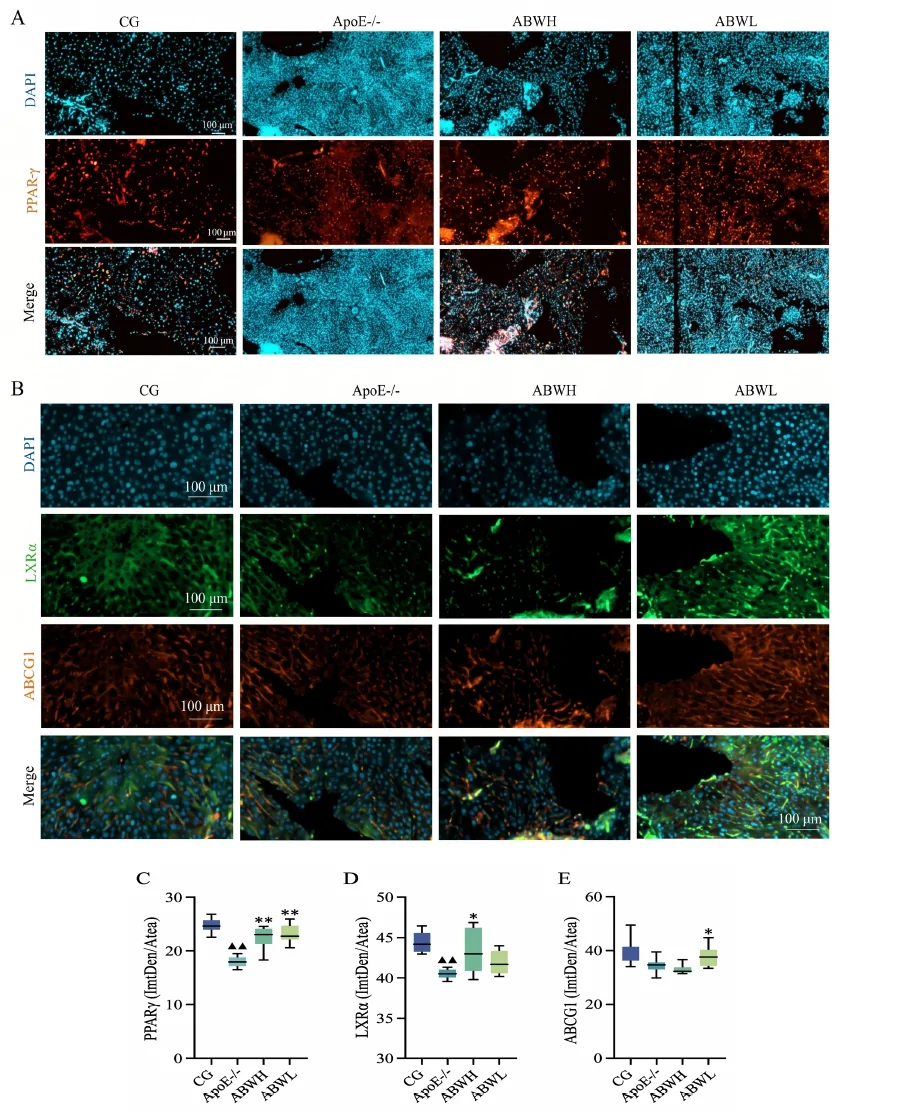
Effect of ABW on the expression of the PPARγ/LXRα pathway in ApoE^-/-^ mice by immunofluorescence staining. (**A**) The immunofluorescence staining of PPARγ (red) at 100× magnification. Nuclei were stained with DAPI. (**B**) The immunofluorescence staining of LXRα (red) and ABCG1 (green) at 200× magnification. Nuclei were stained with DAPI. (**C**) Fluorescence intensity analysis of PPARγ. (**D**) Fluorescence intensity analysis of LXRα expression. (**E**) Fluorescence intensity analysis of the ABCG1 expression. n=6. ^▲^*p* < 0.05, ^▲▲^*p* < 0.01 *vs*. CG. *p < 0.05, **p < 0.01 *vs*. ApoE^-/-^.

**Fig. (5):**
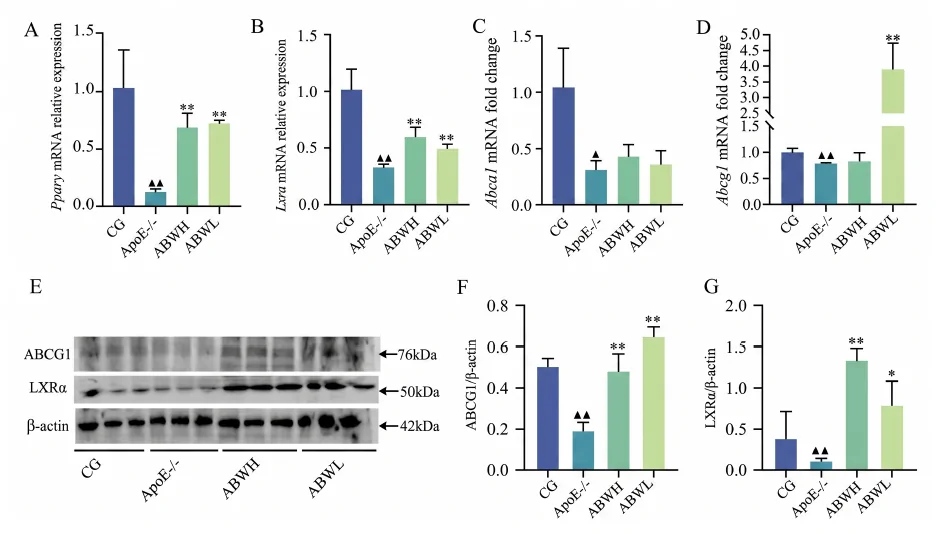
Effect of ABW on the expression of PPARγ/LXRα pathway in ApoE^-/-^mice by qPCR and WB. (**A**) The relative gene expressions of liver PPARγ. (**B**) The relative gene expressions of liver *Lxrα*. (**C**) The relative gene expressions of liver *Abca1*. (**D**) The relative gene expressions of liver*Abcg*1. (**E**) Protein blotting of LXRα and ABCG1 in the study groups. (**F**) Semiquantitative analysis of the ABCG1 expression. (**G**) Semiquantitative analysis of the LXRα expression. n=3. ^▲^*p* < 0.05, ^▲▲^*p* < 0.01 *vs*. CG.*p < 0.05, **p < 0.01 *vs*. ApoE^-/-^.

**Fig. (6):**
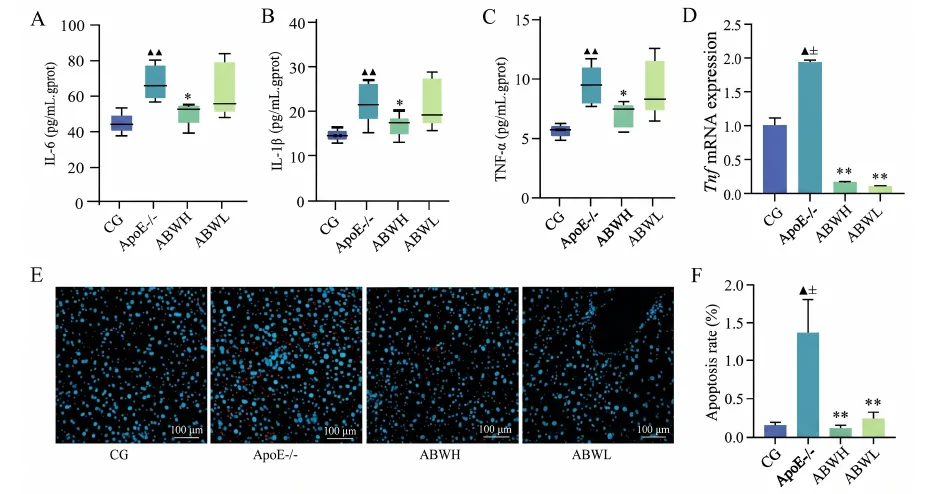
ABW depressed expression of inflammation and apoptosis in the liver. (**A**) The levels of IL-6 in the liver. (**B**) The content of IL-1β in the liver. (**C**) The level of TNF-α. (**D**) The relative gene expressions of *TNF*. (**E**) The apoptosis in the liver (200× magnification). (**F**) The apoptosis rate in the liver. n=6. ^▲^*p* < 0.05, ^▲▲^*p* < 0.01 *vs*. CG.*p < 0.05, **p < 0.01 *vs*. ApoE^-/-^.

**Fig. (7):**
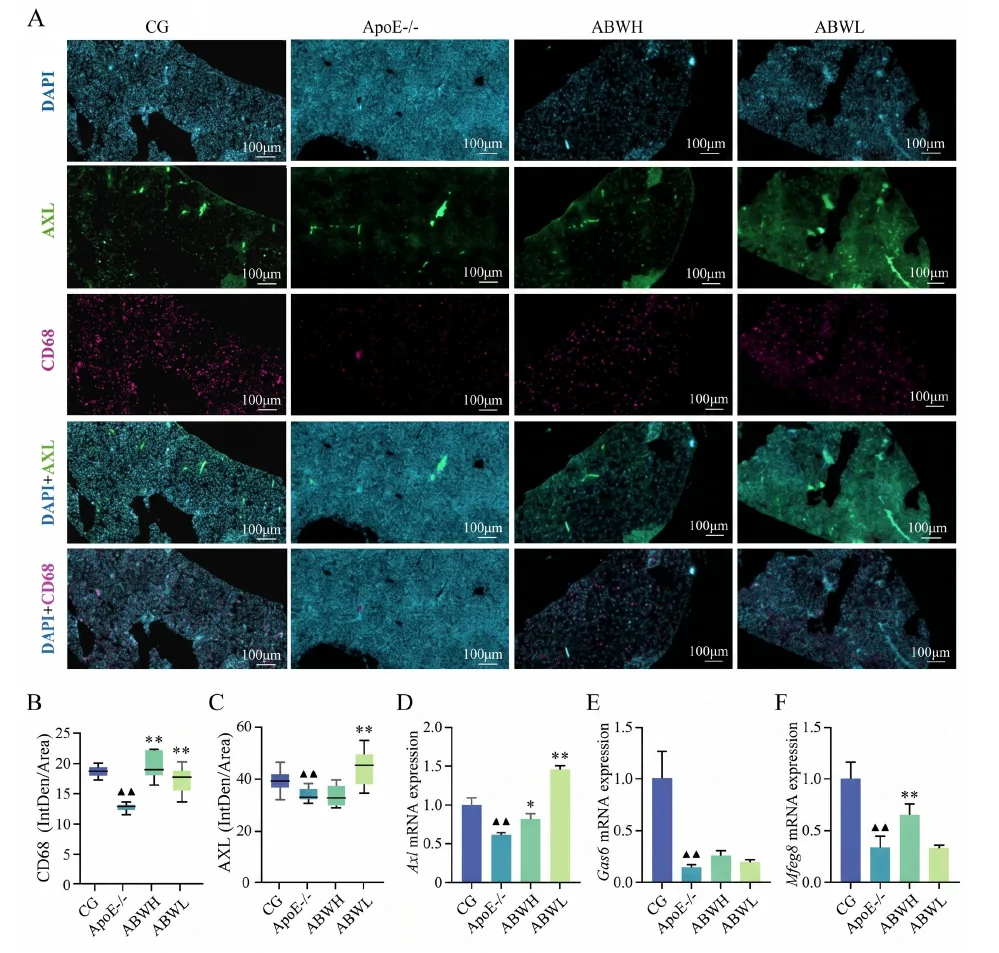
Effect of ABW on the liver protein expression of macrophage efferocytosis in ApoE^-/-^mice. (**A**) The immunofluorescence staining of Cd68 (purple) and Axl (green) at 100× magnification. Nuclei were stained with DAPI. (**B**) Quantitative analysis of Cd68 expression using immunofluorescence. (**C**) Quantitative analysis of Axl expression by immunofluorescence. (**D**) The relative gene expressions of *Axl*. (**E**) The relative gene expressions of *Gas6*. (**F**) The relative gene expressions of *Mfeg8*. ^▲^*p* < 0.05, ^▲▲^*p* < 0.01 *vs*. CG.*p < 0.05, **p < 0.01 *vs*. ApoE^-/-^.

**Fig. (8):**
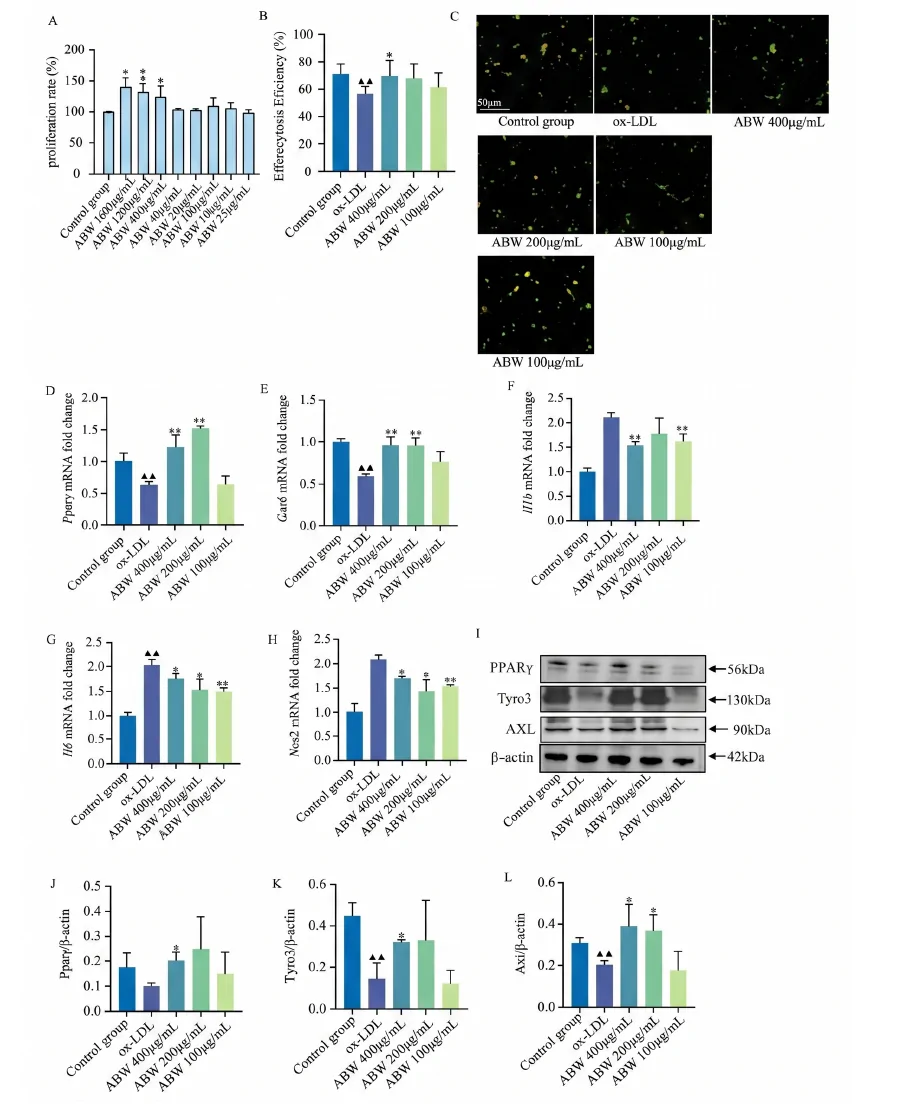
ABW regulated macrophage efferocytosis by the PPARγ pathway. (**A**) Effect of ABW on the proliferation of RAW 264.7 cells. (**B**) Macrophage efferocytosis efficiency (%). (**C**) Representative pictures of macrophage efferocytosis clearing apoptotic neutrophils. (**D**) The relative expression of *Pparγ*. (**E**) The relative expression of *gas6*. (**F**) The relative expression of *Il1b*. (**G**) The relative expression of *IL-6*. (**H**) The relative expression of *Nos2*. (**I**) Relative protein blotting of PPARγ, AXL, and TYRO3. (**J**) Semiquantitative analysis of the PPARγ expression. (**K**) Semiquantitative analysis of the TYRO3 expression. (**L**) Semiquantitative analysis of the AXL expression. n=3. *p < 0.05, **p < 0.01 *vs*. ox-LDL.

**Fig. (9):**
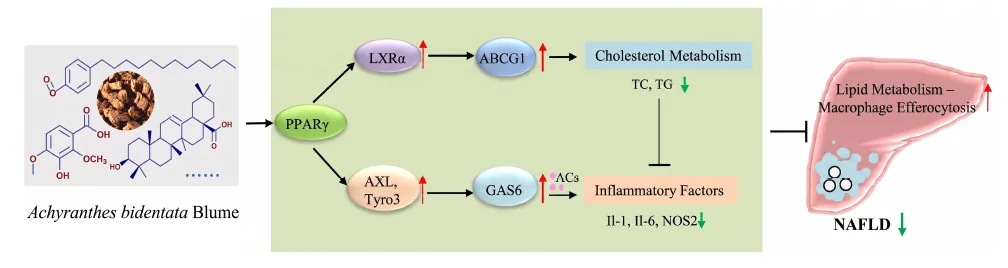
ABW improves NAFLD by activating the PPARγ/LXRα pathway and PPARγ/TAM axis.

**Table 1:** Primer sequences of the gene markers used in the study.

Gene	Forward	Reverse
*Lxrα*	AACTGAAGCGGCAAGAAGAGGAAC	TGGCAGGACTTGAGGAGGTGAG
*Abca1*	AGAAGGAGGCTCGGCTGAAGG	GAGGGATGAGGCTGCTAACAAACC
*Tyro3*	AGCCAGGTGTCCATCTCCATCAG	GCCAGCATCAGACCGTTCCAC
*Gas6*	CCGTTCAGACAGCACCTGGATTG	ACATGCCGTGGTTGATGGTTGG
*Abcg1*	CTGCTGCCTCACCTCACTGTTC	TCTCGTCTGCCTTCATCCTTCTCC
*Megf8*	GCACCTCCTACTCCTCCTGTCTG	ACTCGCCGCTCCCATCATCC
*Pparγ*	GCCAAGGTGCTCCAGAAGATGAC	GTGAAGGCTCATGTCTGTCTCTGTC
*Axl*	GGACCACTGAAGCCACCTTGAAC	GCCACCTTATGCCGATCTACCATG
*β-actin*	GATGGTGGGAATGGGTCAGAAGG	TTGTAGAAGGTGTGGTGCCAGATC

**Table 2:** Characterization of the top 20 compounds in ABW detected by UPLC-MS.

Name	Formula	RT [min]	Ration
D-(+)-Pyroglutamic Acid	C_5_H_7_NO_3_	2.701	10.34
Betaine	C_5_H_11_NO_2_	1.405	9.66
4-Oxoproline	C_5_H_7_NO_3_	2.688	6.45
Citric acid	C_6_H_8_O_7_	2.712	5.76
ethyl 2-{[(hexylamino)carbonyl]amino}-3-phenylpropanoate	C_18_H_28_N_2_O_3_	9.861	3.08
Oleanolic acid	C_30_H_48_O_3_	21.029	2.89
D-(+)-Malic acid	C_4_H_6_O_5_	1.596	2.79
3-Hydroxypicolinic acid	C_6_H_5_NO_3_	2.828	2.61
Ecdysterone	C_27_H_44_O_7_	12.957	2.43
(15Z)-9,12,13-Trihydroxy-15-octadecenoic acid	C_18_H_34_O_5_	16.303	2.33
Oleanolic acid	C_30_H_48_O_3_	18.458	2.32
5-Hydroxymethyl-2-furaldehyde	C_6_H_6_O_3_	5.827	1.61
5-hydroxy-4-methoxy-5,6-dihydro-2H-pyran-2-one	C_6_H_8_O_4_	4.622	1.59
1-Stearoylglycerol	C_21_H_42_O_4_	22.912	1.50
Theophylline	C_7_H_8_N_4_O_2_	1.352	1.46
L-Phenylalanine	C_9_H_11_NO_2_	5.155	1.40
ethyl 4-amino-2-(methylsulfanyl)-1,3-thiazole-5-carboxylate	C_7_H_10_N_2_O_2_S_2_	1.323	1.36
α,α-Trehalose	C_12_H_22_O_11_	1.453	1.01
2-Hydroxyphenylalanine	C_9_H_11_NO_3_	2.064	0.88
Oleanolic acid	C_30_H_48_O_3_	17.898	0.75

**Table 3:** Molecular docking results between ABW active components and PPARγ.

Ingredient	Chemical Structures	Binding Energy	Ingredient	Chemical Structures	Binding Energy
Tetrahydrocor-tisone	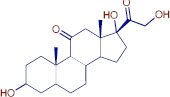	-7.3	(15Z)-9,12,13-Trihydroxy-15-octadecenoic acid	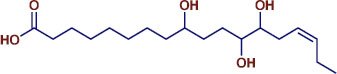	-5.5
Ecdysterone	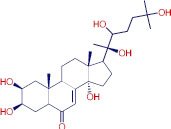	-6.2	Oleanolic acid	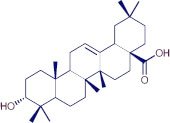	-7.5
4-Dodecylbenzene-sulfonic acid	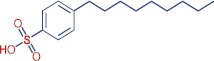	-9.1	Isovanillic acid	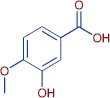	-7.8
Palmitoleic Acid	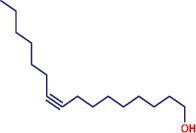	-6.1	5α-Androstan-3,6,17-trione	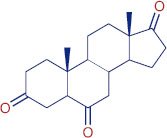	-6.1

## Data Availability

The data supporting the present findings are available from the corresponding author upon a reasonable request.

## LIST OF ABBREVIATIONS

AB: *Achyranthes Bidentata*
ABW: Achyranthes Bidentata Blume Water Extract
ABCA1: ATP-Binding Cassette Transporter A1
ACs: Apoptosis Cells
CD68: Macrophage Antigen CD68
ELISA: Enzyme-Linked Immunosorbent Assay
Gas6: Growth Arrest-Specific 6
LXRα: Liver X Receptor Alpha
MFG-E8: Milk Fat Globule Epidermal Growth Factor 8
NAFLD: Non-Alcoholic Fatty Liver Disease
NF-κB: Nuclear Factor-Kappa Beta
ProS: Protein S
PS: Phosphatidylserine
PPAR: Peroxisome Proliferator-Activated Receptor
PPAR-γ: Peroxisome Proliferator-Activated Receptor Gamma
TC: Cholesterol
TG: Triglyceride
TCM: Traditional Chinese Medicine
TAM: Tyro3, Axl, MerTK
TLR4: Toll-Like Receptor 4
TNF-α: Tumor Necrosis Factor-Alpha
TSA: Tyramide Signal Amplification
